# Development of Plant-Produced Recombinant ACE2-Fc Fusion Protein as a Potential Therapeutic Agent Against SARS-CoV-2

**DOI:** 10.3389/fpls.2020.604663

**Published:** 2021-01-07

**Authors:** Konlavat Siriwattananon, Suwimon Manopwisedjaroen, Phongthon Kanjanasirirat, Priyo Budi Purwono, Kaewta Rattanapisit, Balamurugan Shanmugaraj, Duncan R. Smith, Suparerk Borwornpinyo, Arunee Thitithanyanont, Waranyoo Phoolcharoen

**Affiliations:** ^1^Research Unit for Plant-Produced Pharmaceuticals, Chulalongkorn University, Bangkok, Thailand; ^2^Department of Pharmacognosy and Pharmaceutical Botany, Faculty of Pharmaceutical Sciences, Chulalongkorn University, Bangkok, Thailand; ^3^Department of Microbiology, Faculty of Science, Mahidol University, Bangkok, Thailand; ^4^Excellence Center for Drug Discovery, Faculty of Science, Mahidol University, Bangkok, Thailand; ^5^Department of Microbiology, Faculty of Medicine, Universitas Airlangga, Surabaya, Indonesia; ^6^Institute of Molecular Bioscience, Mahidol University, Salaya, Thailand

**Keywords:** COVID-19, SARS-CoV-2, ACE2-Fc fusion protein, molecular farming, *Nicotiana benthamiana*, plant-produced recombinant protein

## Abstract

Severe acute respiratory syndrome coronavirus 2 (SARS-CoV-2) is the causative agent of coronavirus disease (COVID-19) which has recently emerged as a potential threat to global public health. SARS-CoV-2 is the third known human coronavirus that has huge impact on the human population after SARS-CoV and MERS-CoV. Although some vaccines and therapeutic drugs are currently in clinical trials, none of them are approved for commercial use yet. As with SARS-CoV, SARS-CoV-2 utilizes angiotensin-converting enzyme 2 (ACE2) as the cell entry receptor to enter into the host cell. In this study, we have transiently produced human ACE2 fused with the Fc region of human IgG1 in *Nicotiana benthamiana* and the *in vitro* neutralization efficacy of the plant-produced ACE2-Fc fusion protein was assessed. The recombinant ACE2-Fc fusion protein was expressed in *N. benthamiana* at 100 μg/g leaf fresh weight on day 6 post-infiltration. The recombinant fusion protein showed potent binding to receptor binding domain (RBD) of SARS-CoV-2. Importantly, the plant-produced fusion protein exhibited potent anti-SARS-CoV-2 activity *in vitro*. Treatment with ACE2-Fc fusion protein after viral infection dramatically inhibit SARS-CoV-2 infectivity in Vero cells with an IC_50_ value of 0.84 μg/ml. Moreover, treatment with ACE2-Fc fusion protein at the pre-entry stage suppressed SARS-CoV-2 infection with an IC_50_ of 94.66 μg/ml. These findings put a spotlight on the plant-produced ACE2-Fc fusion protein as a potential therapeutic candidate against SARS-CoV-2.

## Introduction

The coronavirus disease 2019 (COVID-19) outbreak originated in Wuhan, China in late December 2019 (Han et al., 2020; Li et al., 2020; Lupia et al., 2020). The outbreak has spread to more than 200 countries with more than 53.7 million confirmed cases and more than 1.3 million confirmed deaths as of 17 November 2020 (World Health Organization, 2020b). These numbers are still increasing with the ongoing pandemic which overwhelms national health care systems and has had major consequences on global economy. An effective vaccine and treatment are the main priorities to control the pandemic.

COVID-19 is caused by the severe acute respiratory syndrome coronavirus 2 (SARS-CoV-2), which belongs to *Coronaviridae* family. Several members of the family *Coronaviridae* constantly circulate in the human population and usually cause mild respiratory disease (Ciotti et al., 2020; Han et al., 2020). In contrast, the closely related severe acute respiratory syndrome coronavirus (SARS-CoV) and the Middle East respiratory syndrome coronavirus (MERS-CoV) are initially transmitted from animals to humans and cause severe respiratory diseases (Fehr et al., 2017).

SARS-CoV-2 has a single-strand positive-sense RNA genome of approximately 30 kb. The virus comprises four structural proteins, spike (S), nucleocapsid (N), envelope (E), and membrane proteins (M) (Masters, 2006; Amanat and Krammer, 2020; Malik, 2020; Quinlan et al., 2020; Shanmugaraj et al., 2020b). The spike protein is responsible for viral entry into target cells. Entry depends on binding of the receptor binding domain (RBD) on the spike protein to its cellular receptor, which facilitates virus attachment to the receptor and fusion with cell membrane (Li et al., 2005; Masters, 2006; Lei et al., 2020; Quinlan et al., 2020). For SARS-CoV-2, the virus uses the RBD in spike protein to interact with human angiotensin-converting enzyme 2 (ACE2) as a critical initial step to enter into target cells, similar to SARS-CoV (Li et al., 2003; Wong et al., 2004; Hofmann et al., 2005; Shanmugaraj et al., 2020b; Zhang et al., 2020; Zhou et al., 2020b). Therefore, ACE2 has the potential to be used as therapeutic for SARS-CoV-2 infection (Kruse, 2020; Lei et al., 2020).

Our approach for developing SARS-CoV-2 therapeutics focus on transiently producing the human ACE2 protein in plants. Over the last few decades, plants have received considerable attention with advantages of low-cost production, scalability, speed and lack of animal and human pathogens (Phoolcharoen et al., 2011; Shanmugaraj and Ramalingam, 2014; Streatfield et al., 2015; Chan et al., 2016; Rosales-Mendoza et al., 2017). Several potential biologics have been expressed transiently in plants and this is likely to continue with the increasing demand for affordable vaccine (Komarova et al., 2010; Teh et al., 2014). Importantly, plants contain a post-translational modification mechanism which makes them suitable for production of complex proteins, such as antibodies and Fc fusion proteins as described here.

**FIGURE 1:**
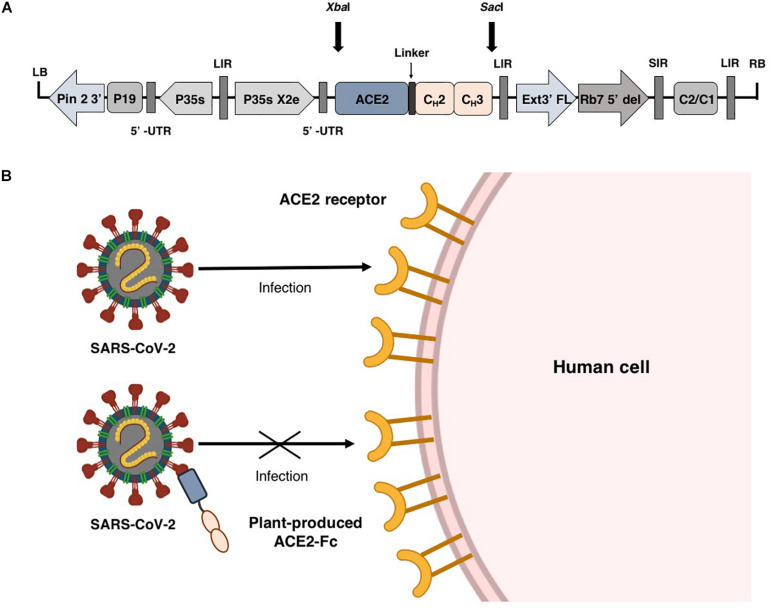
Schematic representation of plant expression vector pBYR2e-ACE2-Fc used in the present study **(A)**. Diagrammatic representation showing the binding of plant-produced ACE2-Fc with SARS-CoV-2 thereby preventing the virus entry into the host cell **(B)**.

Fusion proteins based on the immunoglobulin Fc domain show the ability to facilitate protein expression and enable easy purification of recombinant protein by protein A chromatography (Carter, 2011; Rattanapisit et al., 2019c; Park et al., 2020). Additionally, the Fc domain can also prolong the half-life of the proteins (Cox et al., 2004; Suzuki et al., 2010; Czajkowsky et al., 2012; Kruse, 2020). Several types of Fc fusion proteins had been approved by the FDA (Peters et al., 2010; Powell et al., 2012; Lagassé et al., 2017). Therefore, we engineered ACE2 by fusing N-terminus of the Fc region of human immunoglobulin IgG1 (Figure 1A) and transiently expressed the construct in *Nicotiana benthamiana* using geminiviral vector. The plant produced ACE2-Fc fusion protein was used as a theraputic agent to prevent the attachment of virus to host cell by interacting with SARS-CoV-2 RBD (Figure 1B). Our results showed that the plant-produced ACE2-Fc fusion protein can bind to the RBD and inhibit SARS-CoV-2 infection *in vitro*.

## Materials and Methods

### Plasmid Construction for Expression of ACE2-Fc

The human angiotensin converting enzyme 2 (hACE2) (GenBank accession number: NP_001358344.1) was designed to join with the Fc region of human IgG1 (Genbank accession number: 4CDH_A) by a peptide linker [(GGGGS)_2_] at the C-terminus. The nucleotide sequence of ACE2-Fc construct was optimized for *N. benthamiana* and commercially synthesized by Genewiz, Suzhou, China. The ACE2-Fc sequences were ligated into a geminiviral vector pBYR2eK2Md (pBYR2e) using *Xba*I and *Sac*I restriction sites, at the N-terminus and C-terminus, respectively, to construct the expression vector pBYR2e-ACE2-Fc (Figure 1A).

### Transient Expression of ACE2-Fc in *N. benthamiana* Leaves

The plant expression vector was transformed into *Agrobacterium tumefaciens* GV3101 by electroporation using a MicroPulser (Bio-Rad, United States). The transformants were confirmed by PCR. For ACE2-Fc expression, the *Agrobacterium* pellet containing pBYR2e-ACE2-Fc was resuspended and diluted in 1x infiltration buffer [10 mM 2-(N-morpholino] etanesulfonic acid (MES), 10 mM MgSO_4_, at pH 5.5) to an OD_600_ of 0.2. The suspension was injected into the adaxial side of 6-week-old *N. benthamiana* leaves and maintained at 28°C. For optimization of plant-produced ACE2-Fc, the infiltrated leaves were collected from 3 individual plants on days 2, 4, 6, 8, and 10 after infiltration. Then, we used a vacuum infiltration for large-scale production of ACE2-Fc. The expression level of ACE2-Fc was measured by ELISA assay.

### Protein Extraction and Quantification

The infiltrated leaves were extracted with 1xPBS buffer (phosphate-buffered saline: 137 mM NaCl, 2.68 mMKCl, 10.1 mM Na_2_HPO_4_, 1.76 mM KH_2_PO_4_) at pH 7.4. The suspensions were clarified by centrifugation at 26,000 g for 30 min at 4°C. The supernatants were collected and quantified by an indirect ELISA. 96-well plates (Greiner Bio-One GmbH, Austria) were coated with 50 μl of plant-produced ACE2-Fc or commercial HEK293-produced ACE2-Fc (Abcam, United Kingdom) as a protein standard (diluted in 1xPBS) and plates were incubated overnight at 4°C. The plates were blocked with 5% skim milk powder in 1xPBS for 2 h at 37°C. After blocking, a 1:2,000 dilution of rabbit anti-ACE2 antibody (SinoBiological, United States) in 1xPBS was added into the wells and incubated for 2 h at 37°C. Then, goat anti-rabbit IgG-HRP fusion was added with the dilution of 1:2,000 in 1xPBS (BosterBio, United States) and incubated for 1 h at 37°C. The signal was developed by addition of 50 μl of 3,3′,5,5′-Tetramethylbenzidine (TMB) mixture (Promega, United States) followed by adding 1M H_2_SO_4_. The absorbance was measured at 450 nm using a 96-well plate reader (Molecular Devices, United States). Each sample was loaded in triplicates. Between each step, the plates were washed three times with 1xPBST (1xPBS plus 0.05% Tween-20).

### Protein Purification and Characterization

The total soluble proteins from infiltrated leaves were extracted with 1xPBS pH 7.4 and clarified by centrifugation. The supernatant was filtered by 0.45 μm membrane filter (MilliporeSigma, United States) and loaded onto an affinity chromatography column containing protein-A beads. The column was washed with 1xPBS pH 7.4 followed by 0.1M glycine, pH 2.7 for ACE2-Fc elution. The elution sample was instantly neutralized with the addition of 1.5M Tris-HCl, pH 8.8. The purified plant-produced ACE2-Fc was analyzed by using sodium dodecyl sulfate-polyacrylamide gel electrophoresis (SDS-PAGE) and western blotting under non-reducing and reducing conditions using commercial HEK293-produced ACE2-Fc fusion protein as a positive control. The plant-produced ACE2-Fc samples were separated on 8% SDS-PAGE and the gel was stained with Coomassie brilliant blue. For western blot analysis, proteins were transferred to a nitrocellulose membrane (Biorad, United States). The membrane was blocked using 5% skim milk in 1xPBS and separately probed with ACE2-specific antibody using a rabbit anti-ACE2 antibody (SinoBiological, United States) followed by goat anti-rabbit-HRP fusion (BosterBio, United States) and Fc domain-specific antibody using an anti-human Gamma chain-HRP fusion (The Binding Sites, United Kingdom) with the dilution of 1:2,000 in 1xPBS. The membrane was washed with 1xPBST and the signal developed using an ECL reagent (Abcam, United Kingdom).

### SARS-CoV-2 RBD Binding by ELISA

The binding activity of the purified plant-produced ACE2-Fc to SARS-CoV-2 RBD was analyzed by ELISA. 96-well plate (Greiner Bio-One GmbH, Austria) was coated with 100 ng of plant-produced ACE2-Fc and incubated overnight at 4°C. The wells were blocked using 5% skim milk in 1xPBS for 2 h at 37°C. The plate was washed three times with 1xPBST and incubated with various dilutions of proteins including the RBD of SARS-CoV-2 produced from Sf9 cells (Genscript Biotech, United States), S1 protein of porcine epidemic diarrhea virus (PEDV) (Supplementary Figure S1), and PBS as negative controls for 2 h at 37°C. After washing, an anti-6X His tag-HRP fusion (Abcam, UK) diluted in 1xPBS was added into the wells and incubated for 2 h at 37°C. For detection, a TMB solution (Promega, United States) was added into the plate. The enzymatic reactions were stopped by adding 1M H_2_SO_4_. The absorbance at 450 nm was measured using a 96-well microplate reader (Molecular Devices, United States).

### *In vitro* Antiviral Assay

A total of 10,000 Vero E6 cells were cultured in a 96-well plate (Corning, United States) overnight at 37°C in a 5%CO_2_ atmosphere. For the post-treatment condition, 25TCID_50_ (50% tissue culture infective dose) of SARS-CoV-2 was adsorbed for 2 h at 37°C, after washing the cells with 1xPBS, fresh culture medium (DMEM with 2%FBS) was added. Various concentrations of ACE2-Fc were directly added to the culture medium and cells were maintained at 37°C in a 5%CO_2_ incubator for 48 h. For pre-entry treatment, a mixture of ACE2-Fc and 25TCID_50_ of SARS-CoV-2 was incubated at 37°C for 1 h before viral adsorption for 2 h. The cells were washed twice with 1xPBS followed by the addition of fresh culture medium (DMEM with 2%FBS) after which cells were maintained under standard conditions for an additional 48 h. Positive convalescent serum (heat-inactivated for 30 min at 56°C) of a COVID-19 patient and an anti-human IgG-FITC antibody (Santa Cruz Biotechnology, United States) were used as positive and negative controls, respectively. The experiment was performed in duplicates. A high-content imaging system was used for the detection of the SARS-CoV-2 nucleocapsid. The cells in the 96-well plate were fixed and permeabilized with 50% (v/v) acetone in methanol on ice for 20 min washed once with 1xPBST with 0.5% Tween detergent, followed by blocking in 1xPBST with 2% (w/v) BSA for 1 h at room temperature. After blocking, the cells were incubated with a 1:500 dilution of a rabbit monoclonal primary antibody specific for the SARS-CoV nucleoprotein (NP) (SinoBiological, United States) for 1 h at 37°C. The unbound antibody was removed by washing with 1xPBST thrice. Then, cells were incubated with a 1:500 dilution of an Alexa Fluor 488 conjugated goat anti-rabbit IgG (H + L) highly cross-adsorbed secondary antibody. Nuclei of the cells were stained with Hoechst dye (Thermo Fisher Scientific, United States). The fluorescent signals were detected and analyzed using a high-content imaging system (PerkinElmer, United Kingdom) at 40x magnitude. The percentage of infected cells in each well was automatically obtained randomly from 13 images per well using Harmony software (PerkinElmer, United Kingdom).

## Results

### Transient Expression of ACE2-Fc Fusion Protein in *N. benthamiana* Plants

**FIGURE 2:**
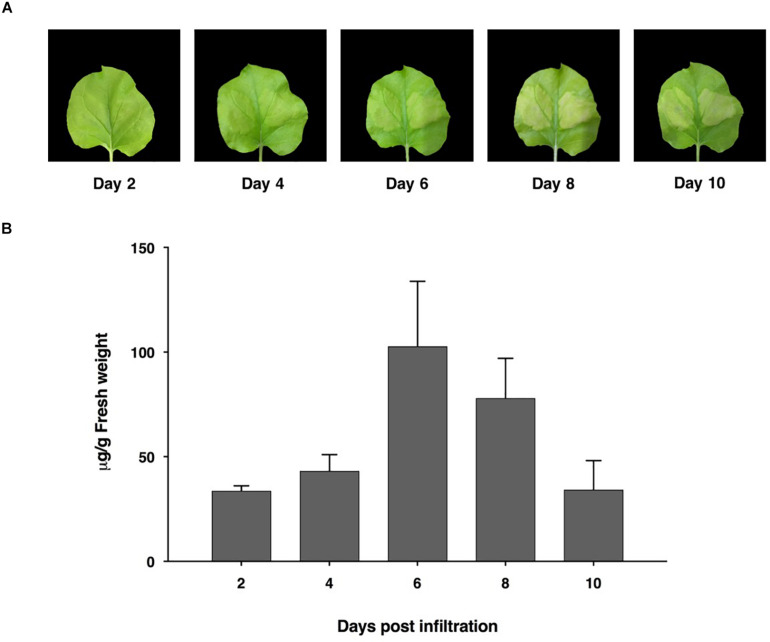
Expression profiles of ACE2-Fc in *N. benthamiana* leaves on days 2, 4, 6, 8, and 10 after agroinfiltration. Leaf necrosis **(A)** Quantification of plant-produced ACE2-Fc **(B)**. The infiltrated leaves were collected from 3 individual plants in each day post infiltration. Data were analyzed by indirect ELISA assay using ACE2-specific antibody and presented as mean ± SD of triplicates.

The ACE2-Fc fusion protein gene was designed using codon preferred for *N. benthamiana* and commercially synthesized. We generated the ACE2-Fc fusion protein by fusing the ACE2 protein to the N terminus of the Fc region. To produce the ACE2-Fc fusion protein in plants, the ACE2-Fc gene was incorporated into the pBYR2e geminiviral vector (Chen et al., 2011; Rattanapisit et al., 2019a, b) and subsequently introduced into *N. benthamiana* plants through agroinfiltration. The expression of the ACE2-Fc fusion protein induced mild necrosis in leaves (Figure 2A). The protein was expressed highest on day 6 post-infiltration, with up to 100 μg/g leaf fresh weight (Figure 2B).

### Purification of ACE2-Fc Fusion Protein From *N. benthamiana* Leaves

**FIGURE 3:**
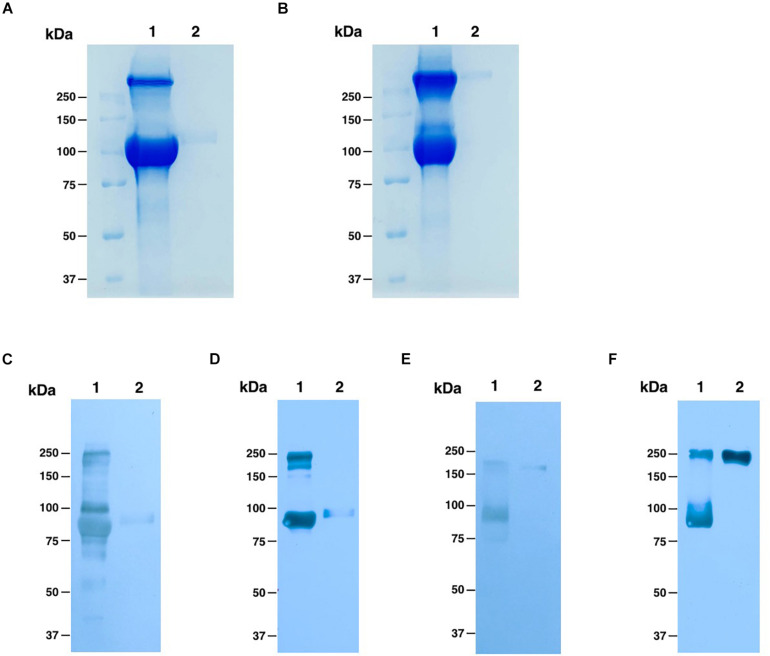
Analysis of purified plant-produced ACE2-Fc (lane 1) and commercial HEK293-produced ACE2-Fc (lane 2). Coomassie-stained SDS-PAGE under reducing **(A)** and non-reducing conditions **(B)**. Western blotting analysis under reducing condition with detection using a rabbit anti-ACE2 antibody **(C)** and an anti-human gamma-HRP conjugated antibody **(D)**. Western blotting analysis under non-reducing condition probed with a rabbit anti-ACE2 antibody **(E)** and an anti-human gamma-HRP conjugated antibody **(F)**.

To purify the ACE2-Fc fusion protein, we used one-step protein A affinity chromatography. We estimate that the plant-produced ACE2-Fc fusion protein was ∼90% pure based on visual inspection of a Coomassie blue stained gel, with a molecular weight of around 100 kDa under reducing condition, which had no different molecular weight comparing to commercial HEK293-prduced ACE2-Fc protein (Figure 3A), while we could observe the protein dimer of both ACE2-Fc fusion proteins under non-reducing condition, which showed the protein size at 250 kDa (Figure 3B). The folding of plant-produced ACE2-Fc fusion protein was confirmed by western blot analysis using ACE2-specific and Fc domain-specific antibodies. The results indicated that purified plant-produced ACE2-Fc proteins showed two major bands at the molecular weight of approximately 100 and 250 kDa under reducing (Figures 3C,D) and non-reducing condition (Figures 3E,F), respectively, which were same as the profiles on Coomassie blue stained gel.

### Binding of ACE2-Fc With RBD of SARS-CoV-2

**FIGURE 4:**
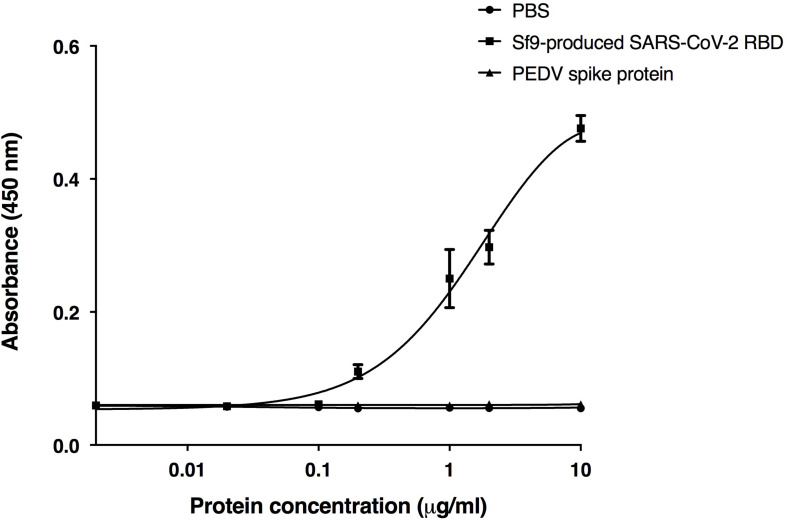
Binding activity of the plant-produced ACE2-Fc with the commercial receptor binding domain of SARS-CoV-2 (SARS-CoV-2 RBD) from Sf9 cells was analyzed by ELISA. PBS buffer and S1 protein of PEDV were used as negative controls. Data are presented as mean ± SD of triplicates.

The receptor binding domain (RBD) of SARS-CoV-2 was previously shown to bind to the human ACE2 receptor on host cells (Rabi et al., 2020; Shanmugaraj et al., 2020b; Yuki et al., 2020; Zhou et al., 2020a). We used RBD binding assay to confirm the *in vitro* binding activity of the plant-produced ACE2-Fc fusion protein. The purified ACE2-Fc fusion protein was immobilized in the wells of a microtiter plate. Eight different dilutions of the commercial Sf9-produced RBD protein of SARS-CoV-2, the S1 protein of the porcine epidemic diarrhea virus (PEDV), and PBS were added into the ELISA plate with triplicate wells. The results (Figure 4) showed that the plant-produced ACE2-Fc fusion protein produced substantially high OD signals with the RBD of SARS-CoV-2, compared to the negative PBS control and the PEDV S1 protein. Our data are consistent with the binding of the RBD of SARS-CoV-2 to the plant-produced ACE2-Fc fusion protein.

### Anti-SARS-CoV-2 Activity of the Plant-Produced ACE2-Fc Fusion Protein

**FIGURE 5:**
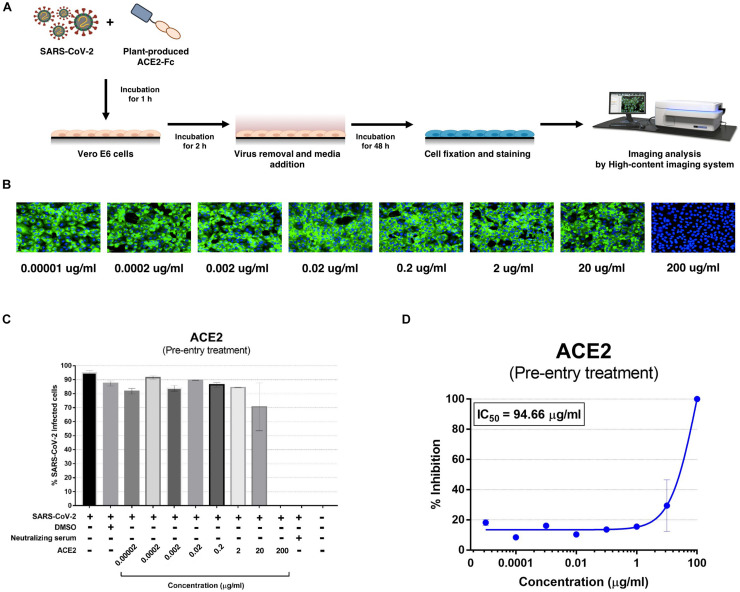
Dose-dependent effect of plant-produced ACE2-Fc on SARS-CoV-2 inhibition and neutralization at the pre-infection phase. Experimental design of plant-produced ACE2-Fc and SARS-CoV-2 mixture added to Vero E6 cells (at 25TCID_50_) **(A)**. SARS-CoV-2 infection profiles in Vero E6 cells which were treated with eight concentrations of plant-produced ACE2-Fc **(B)**. Percentage of SARS-CoV-2 inhibition in Vero E6 cells, which were treated with eight concentrations of plant-produced ACE2-Fc starting with 200 μg/ml **(C)**. Efficacy of SARS-CoV-2 inhibition in Vero E6 cells, which were treated by eight concentrations of plant-produced ACE2-Fc **(D)**. The data were showed as mean ± SD of triplicates in individual concentrations.

**FIGURE 6:**
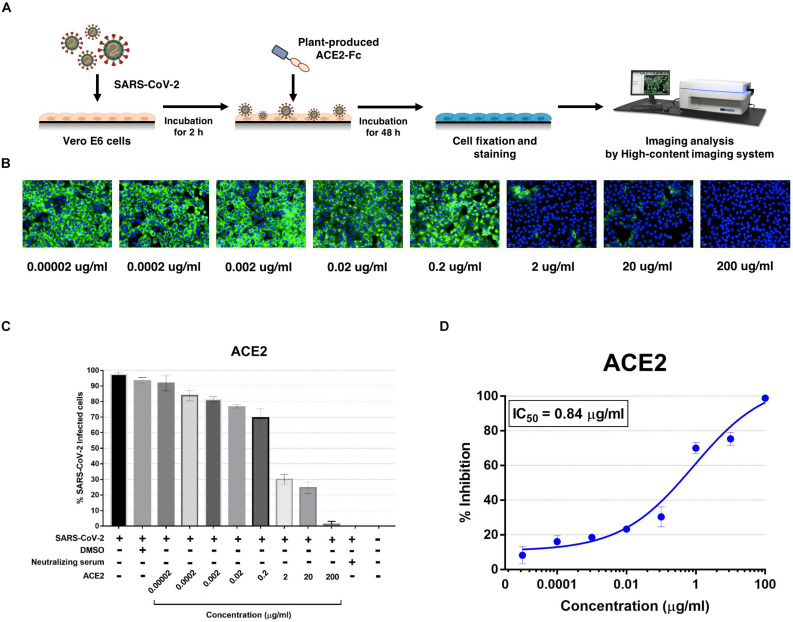
Dose-dependent effect of plant-produced ACE2-Fc on SARS-CoV-2 inhibition and neutralization at the post-infection phase. Experimental design of plant-produced ACE2-Fc and SARS-CoV-2 mixture added to Vero E6 cells (at 25TCID_50_) **(A)**. SARS-CoV-2 infection profiles in Vero E6 cells which were treated with eight concentrations of plant-produced ACE2-Fc **(B)**. Percentage of SARS-CoV-2 inhibition in Vero E6 cells, which were treated with eight concentrations of plant-produced ACE2-Fc starting with 200 μg/ml **(C)**. Efficacy of SARS-CoV-2 inhibition in Vero E6 cells, which were treated by eight concentrations of plant-produced ACE2-Fc **(D)**. The data were showed as mean ± SD of triplicates in individual concentrations.

The plant-produced ACE2-Fc fusion protein was tested for anti-SARS-CoV-2 activity at the pre- and post-entry phases. For the pre-entry treatment, the protein was pre-incubated with SARS-CoV-2 at 37°C for 1 h before inoculation onto Vero cells. Viral adsorption was undertaken for 2 h in the presence of the protein before removing any unbound virus. The cells were cultured for 48 h before harvesting for analysis (Figure 5A). The ACE2-Fc fusion protein in pre-entry treatment showed lower efficiency of SARS-CoV-2 inhibition in Vero cells (Figures 5B,C). The IC_50_ of the plant-produced ACE2-Fc fusion protein for the pre-entry treatment was measured by the percentage of SARS-CoV-2 inhibition curve with 94.66 μg/ml (Figure 5D). For the post-entry treatment, Vero cells were inoculated with SARS-CoV-2 for 2 h. After washing, the protein was added and cells were incubated for 48 h before harvesting for analysis (Figure 6A). The results showed that the post-treatment inhibited SARS-CoV-2 infection at the concentration starting with 1 μg/ml (Figures 6B,C). The IC_50_ for post-entry treatment was 0.84 μg/ml (Figure 6D). The serum from COVID-19 patient and an anti-human IgG were used as the positive and negative controls, respectively, for both pre- and post-treatment experiments.

## Discussion

The COVID-19 outbreak caused by the novel betacoronavirus SARS-CoV-2 is responsible for an ongoing pandemic which is having an unprecedented impact on the human population with millions of infected cases. The virus has spread rapidly through human-to-human transmission and has affected numerous countries around the world and has emerged as a significant threat to public health, the global economy and society (Shanmugaraj et al., 2020b).

The World Health Organization (WHO) has declared COVID-19 as pandemic and a public health emergency of international concern (World Health Organization, 2020a). Although COVID-19 pathogenesis has still not been completely elucidated, early reports showed that SARS-CoV-2 binds to the host cell receptor angiotensin-converting enzyme-2 (ACE2) through the RBD domain in the spike (S) protein to infect human epithelial cells in alveoli, which can cause a cytokine storm resulting in respiratory failure and ultimately death. The virus can infect cells of the lungs, kidneys, heart and intestine, resulting in organ damage leading to multiple organ dysfunction syndrome (MODS) (Sun et al., 2020; Xie et al., 2020). Currently there are no approved antiviral drugs or vaccines for COVID-19. Reducing the mortality rate amongst COVID-19 infected patients is a primary goal, as is controlling the rapid spread of the infection by developing therapeutic and preventive strategies.

Recently, rapid progress has been made with diagnostic kits/reagents, drug repurposing, immunotherapeutic strategies and vaccine development in response to the COVID-19 pandemic. The scientific communities in almost all countries are in the race to develop an effective and safe vaccine against SARS-CoV-2. Many molecular targets are considered as potential candidates to combat COVID-19 (Le et al., 2020) including recombinant ACE2 as it has shown to have therapeutic potential for SARS-CoV, and is known to protect against severe acute lung injury (Huentelman et al., 2005; Imai et al., 2005; Kuba et al., 2005; Zou et al., 2014). ACE2 is the *in vivo* SARS-CoV-2 functional receptor expressed by epithelial cells of many organs such as the lung, intestine, kidney, and blood vessels (Hamming et al., 2004). Recombinant ACE2 (rACE2) was reported to have a short half-life and fast clearance rate in contrast to a rACE2 with a Fc fusion protein (rACE2-Fc) (Liu et al., 2018). Lei et al. (2020) showed that the ACE2-Fc fusion protein can able to neutralize both SARS-CoV and a SARS-CoV-2 pseudovirus *in vitro* (Lei et al., 2020).

In recent years, plants have been utilized for the production of recombinant biopharmaceuticals and vaccine candidates for several human and veterinary diseases. Many reports have shown the potential of plant expression systems for the rapid production of biopharmaceuticals and proven that plant produced recombinant proteins are as effective as the mammalian cell-produced counterparts in producing neutralizing antibodies against a particular pathogen or infection (Koya et al., 2005; Thanavala et al., 2005; Huang et al., 2008; Kanagarajan et al., 2012; Dolleweerd et al., 2014; Vamvaka et al., 2016; Shanmugaraj et al., 2020a). Moreover, plant biopharming provides the economical production of desirable biopharmaceuticals without high investment costs compared to other available industrial facilities using a fermentation system (Twyman et al., 2003; Ko and Koprowski, 2005; Basaran and Rodríguez-Cerezo, 2008; Shinmyo and Kato, 2010; Fischer et al., 2013; Stoger et al., 2014; Yao et al., 2015; Burnett and Burnett, 2019; Margolin et al., 2020). The production costs in plant biopharming processes are commonly 0.1% of mammalian cell-based technologies and 2–10% of bacterial expression systems (Yao et al., 2015). Due to the fact that plant-made biopharmaceuticals provide a cost-effective alternative to protect against emerging infectious diseases, in this study, we demonstrated the feasibility of using a plant expression system to transiently express an ACE2-Fc fusion protein that could be useful to develop affordable antiviral treatment against SARS-CoV-2. The biological activity of the plant produced ACE2-Fc fusion protein was characterized *in vitro*.

The ACE2-Fc fusion protein was codon optimized and cloned into the geminiviral vector pBYR2e for plant expression, and the recombinant fusion protein was transiently expressed in *N. benthamiana* plants. Our results showed that the ACE2-Fc fusion protein can be produced in a large scale in *N. benthamiana* in less than 10 days after the construction of the plant expression vector. Recent advancements in plant protein production strategies through using deconstructed viral vector systems and transient expression has reduced the protein production timeline in contrast to stable expression systems which requires several months for recombinant protein production. The safety, scalability and robustness of the plant transient expression system have proved the commercial viability of the system (Sainsbury and Lomonossoff, 2014; Canto, 2016; Park and Wi, 2016). We showed that transiently expressing an ACE2-Fc fusion protein by using a geminiviral vector produced yields of up to 100 μg/g leaf fresh weight in *N. benthamiana* leaves with one-step protein A affinity chromatography. However, additional purification is required in order to meet the quality standards for commercial use. We then investigated the biological activity of the plant-derived ACE2-Fc fusion protein *in vitro.* The plant-produced ACE2-Fc fusion protein exhibits potent binding to the Sf9-produced RBD protein of SARS-CoV-2. The results from the antiviral assay demonstrated a potent inhibitory effect of the ACE2-Fc fusion protein against SARS-CoV-2. The IC_50_ of ACE2-Fc fusion protein was 94.66 and 0.84 μg/ml for the pre-entry and post-infection, respectively. The ACE2-Fc fusion protein showed better inhibition in the post-infection treatment (Figure 6), as compared to the pre-entry treatment (Figure 5).

ACE2 is found in many organs and ACE2 variants are reported to protect from acute respiratory distress syndrome (Imai et al., 2008) and kidney disease (Wysocki et al., 2010) by acting as a negative regulator of the renin angiotensin system (Kuba et al., 2010). Recently, ACE2 has been the focus of a rational therapeutic design against COVID pandemic. ACE2 was reported as the major receptor for SARS and SARS-CoV-2 wherein the virus binds to the ACE2 cell receptor and enters host cells resulting in severe lung injuries. Earlier reports have shown the therapeutic potential of rACE2 for SARS-CoV (Kuba et al., 2005) and its ability to protect from severe acute lung failure (Gu et al., 2016). Apeiron biologics is currently running a pilot human trial in China to investigate the potential of their drug candidate APN01, recombinant human ACE2 for use as a therapeutic agent for the treatment of COVID-19. Here we have shown for the first time that a plant-produced ACE2-Fc fusion protein has the potential to be developed as a therapeutic agent, alone or in combination with other therapeutic agents or with vaccines for the treatment of COVID-19.

## Conclusion

The rapid global spread of SARS-CoV-2 emphasizes the urgent need for the development of effective vaccines and therapeutics that are affordable and scalable. Although studies have shown structural similarities between the RBD of SARS-CoV and SARS-CoV-2, known neutralizing monoclonal antibodies against SARS-CoV might not neutralize SARS-CoV-2 (Rattanapisit et al., 2020; Tian et al., 2020). Hence, it is necessary to develop specific vaccines or treatment strategies to treat SARS-CoV-2 infection. Here we showed that a plant expression system can rapidly and effectively produce a functional ACE2-Fc fusion protein on a large scale. Moreover, the plant produced ACE2-Fc fusion protein exhibits anti-SARS-CoV-2 activity in post-entry treatment *in vitro* suggesting it could be used as post-exposure therapeutic to treat COVID-19. However, further progress toward the goal of establishing an affordable therapeutic intervention program requires animal studies to confirm the efficacy and safety of the plant produced protein against SARS-CoV-2.

## Data Availability Statement

The original contributions presented in the study are included in the article/Supplementary Material, further inquiries can be directed to the corresponding author/s.

## Author Contributions

DS, AT, and WP designed all experiments. KS, KR, and BS performed protein expression, protein purification, and ELISA. SM, PK, PB, and SB performed the anti-viral assay. All authors analyzed the data and contributed to manuscript preparation.

## Conflict of Interest

WP from Chulalongkorn University is a founder/shareholder of Baiya Phytopharm Co., Ltd. This study was funded by BaiyaPhytopharm Co., Ltd. The remaining authors declare that the research was conducted in the absence of any commercial or financial relationships that could be construed as a potential conflict of interest.
