# Supplementary material for: Development of Plant-Produced Recombinant ACE2-Fc Fusion Protein as a Potential Therapeutic Agent Against SARS-CoV-2
Source: Front Plant Sci. 2021 Jan 7;11:604663. doi: 10.3389/fpls.2020.604663 (PMC7874119; doi:10.3389/fpls.2020.604663)
Supplement: Supplementary file 1 [file Image_1.pdf]

Plant-produced recombinant ACE2-Fc fusion protein as anti-SARS-CoV-2 agent

Konlavat Siriwattananon[1,2], Suwimon Manopwisedjaroen[3], Phongthon Kanjanasirirat[4], Priyo Budi Purwono[3,5], Kaewta Rattanapisit[1,2], Balamurugan Shanmugaraj[1,2] Duncan R. Smith[6], Suparerk Borwornpinyo[4], Arunee Thitithanyanont[3], Waranyoo Phoolcharoen[1, 2]*

[1] Research unit for Plant-produced Pharmaceuticals, Chulalongkorn University, Bangkok, Thailand

[2] Department of Pharmacognosy and Pharmaceutical Botany, Faculty of Pharmaceutical Sciences, Chulalongkorn University, Bangkok, Thailand.

[3] Department of Microbiology, Faculty of Science, Mahidol University, Bangkok, Thailand

[4] Excellence Center for Drug Discovery (ECDD), Faculty of Science, Mahidol University, Bangkok 10400 Thailand

[5] Department of Microbiology, Faculty of Medicine, Universitas Airlangga, Surabaya, Indonesia

[6] Institute of Molecular Bioscience, Mahidol university, Salaya, Nakhon Prathom, Thailand

*Correspondence: Waranyoo.P@chula.ac.th; Tel: 662-218-8359; Fax: 662-218-8357

Figure S1. Expression of spike protein of porcine epidemic diarrhea virus (PEDV) in *N. benthamiana*. Schematic representation showing the plant expression construct pBYR2e PEDV S1-His used (A). Diagrammatic representation showing the overview of transient expression of PEDV S1 protein in *N. benthamiana* (B). Western blotting of plant-produced PEDV S1 under reducing conditions. SARS-CoV-2 RBD-His from Sf9 cells (Genscript Biotech, USA) (lane 1 – positive control) and purified plant-produced PEDV S1-His (lane 2) probed with anti-His-HRP conjugate antibody. The red and black arrows indicate the presence of S1-His of PEDV and SARS-CoV-2 RBD-His, respectively (C).

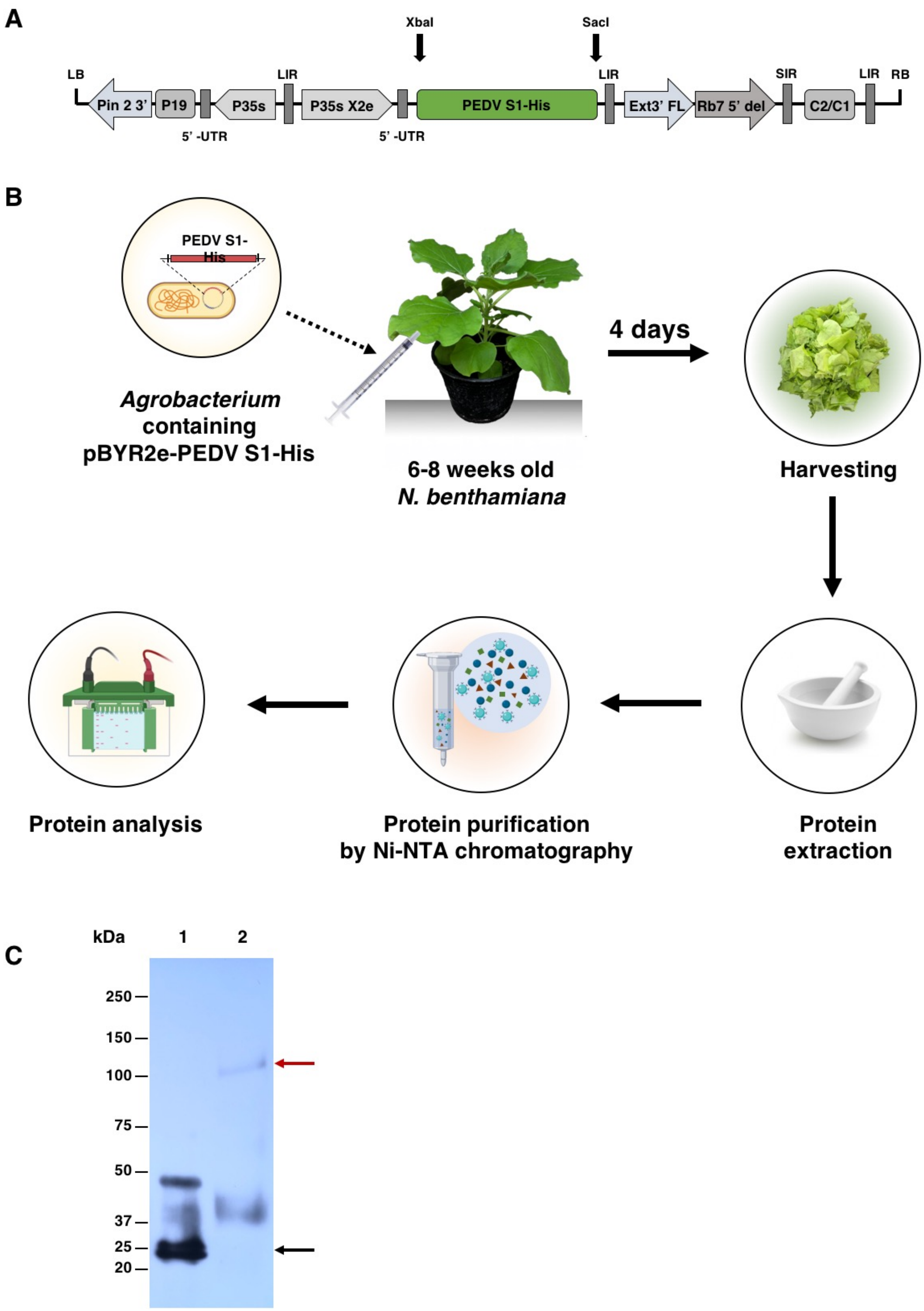
A
XbaI
SacI
LB
Pin 2 3'
P19
5' -UTR
P35s
LIR
P35s X2e
5' -UTR
PEDV S1-His
LIR
Ext3' FL
Rb7 5' del
SIR
C2/C1
LIR
RB
B
PEDV S1-His
Agrobacterium containing pBYR2e-PEDV S1-His
6-8 weeks old *N. benthamiana*
4 days
Harvesting
Protein extraction
Protein purification by Ni-NTA chromatography
Protein analysis
C
kDa
1
2
250
150
100
75
50
37
25
20
